# Rhizoma Paridis saponins attenuate Gram‐negative bacteria‐induced inflammatory acne by binding to KEAP1 and modulating Nrf2 and MAPK pathways

**DOI:** 10.1111/jcmm.18146

**Published:** 2024-03-01

**Authors:** Yang Yang, Chaofan Wang, Juan Wang, Lingli Yang, Zheng Lv, Quan An, Yiming Wang, Xue Shao, Fei Wang, Tong Huo, Jiali Liu, Haoshu Luo, Qianghua Quan

**Affiliations:** ^1^ R&D Department Yunnan Baiyao Group Health Products Co., Ltd Kunming Yunnan China; ^2^ R&D Department East Asia Skin Health Research Center Beijing China; ^3^ R&D Department Yunnan Baiyao Group Shanghai Science & Technology Co., Ltd Shanghai China; ^4^ State Key Laboratory of Animal Biotech Breeding, College of Biological Sciences China Agricultural University Beijing China; ^5^ Key Laboratory for Space Bioscience and Biotechnology, School of Life Sciences Northwestern Polytechnical University Xi'an Shaanxi China

**Keywords:** acne vulgaris, anti‐inflammation, Gram‐negative bacteria, kelch‐like ECH‐associated protein 1, mitogen‐activated protein kinase, polyphyllin H, Rhizoma Paridis

## Abstract

Acne vulgaris represents a chronic inflammatory condition, the pathogenesis of which is closely associated with the altered skin microbiome. Recent studies have implicated a profound role of Gram‐negative bacteria in acne development, but there is a lack of antiacne agents targeting these bacteria. Polyphyllins are major components of Rhizoma Paridis with great anti‐inflammatory potential. In this study, we aimed to evaluate the antiacne effects and the underlying mechanisms of PPH and a PPH‐enriched Rhizoma Paridis extract (RPE) in treating the Gram‐negative bacteria‐induced acne. PPH and RPE treatments significantly suppressed the mRNA and protein expressions of interleukin (IL)‐1β and IL‐6 in lipopolysaccharide (LPS)‐induced RAW 264.7 and HaCaT cells, along with the intracellular reactive oxygen species (ROS) generation. Furthermore, PPH and RPE inhibited the nuclear translocation of nuclear factor kappa‐B (NF‐κB) P65 in LPS‐induced RAW 264.7 cells. Based on molecular docking, PPH could bind to kelch‐like ECH‐associated protein 1 (KEAP1) protein. PPH and RPE treatments could activate nuclear factor erythroid 2‐related factor 2 (NRF2) and upregulate haem oxygenase‐1 (HO‐1). Moreover, RPE suppressed the mitogen‐activated protein kinase (MAPK) pathway. Therefore, PPH‐enriched RPE showed anti‐inflammatory and antioxidative effects in vitro, which is promising for alternative antiacne therapeutic.

## 1 INTRODUCTION

Acne vulgaris is a worldwide distributed skin inflammatory disease, which could affect from 20% to 95% of individuals in several countries.^1^ Inflammatory acne lesions (papules, pustules and nodules) have a significant impact on mental health and social relationships, along with physical consequences including a high risk of permanent scarring and prolonged hyperpigmentation.^1^, ^2^, ^3^ It was extensively suggested that the overaccumulation of the sebum, follicular hyperkeratinization, excessive colonization of *Propionibacterium acnes* within the pilosebaceous and inflammation processes are all involved in the pathogenesis of acne.^4^, ^5^ In this regard, proinflammatory cytokines induce hyperkeratinization in the infundibulum and sebaceous duct, leading to comedogenesis.^6^ Moreover, the inflammatory mediators (such as interleukin [IL]‐1 and IL‐6) secreted from keratinocytes are responsible for macrophages activation and inflammation exacerbation with an overproduction of cytokines and reactive oxygen species (ROS).^6^ Therefore, the development of acne can be attributed to the inflammatory response from various skin cell types. However, the anti‐inflammatory properties of saponins were mostly evaluated in individual cell types.

The skin microbiome consists of various Gram‐positive and Gram‐negative bacterial phyla.^7^ The overgrowth of *P*. *acnes*, which is a Gram‐positive bacterium, was demonstrated to be the leading stimulating factor for inflammatory acne.^4^, ^5^ Recent studies utilized advanced culture‐independent methods have reported that there is no statistically significant difference in the abundance of *P*. *acnes* between acne patients and healthy individuals.^8^, ^9^ These findings challenge the previously standpoints that *P*. *acnes* is the primary causative agent of acne. Conversely, Gram‐negative bacteria were found positively correlated with acne and folliculitis of facial skin.^8^ Additionally, the excessive colonization of Pseudomonadaceae, a Gram‐negative bacterium, was found to contribute to the pathogenesis of swimmer acne.^8^, ^9^ Lipopolysaccharide (LPS) is a representative pathogen‐associated molecular pattern (PAMP) component of Gram‐negative bacteria. Recognition of LPS by Toll like receptor‐4 (TLR4) triggers downstream inflammatory pathways.^10^ All together, these findings suggest the role of Gram‐negative bacteria in acne exacerbation has been underestimated.

Drugs with antibacterial and anti‐inflammatory properties are commonly used in the treatment of acne.^6^ Activation of the nuclear factor kappa‐B (NF‐κB) pathway and mitogen‐activated protein kinase (MAPK) pathway are responsible for the upregulation of the expression of proinflammatory factors, such as IL‐1β and IL‐6.^3^, ^4^ In addition, oxidative stress induced by the excessive production of sebum in pilosebaceous and thus contribute to the development of inflammatory acne. As a result, agents that act suppressing the ROS, IL‐1β and IL‐6 productions have been widely used for acne treatment.^4^, ^6^ Nuclear factor erythroid 2‐related factor 2 (NRF2) is a key regulator of cellular response against oxidative stress and inflammation by upregulating antioxidant factors and by modulating NF‐κB nuclear translocation.^11^, ^12^ The Kelch‐like ECH‐associated protein 1 (KEAP1) protein negatively regulate NRF2 by forming the NRF2‐KEAP1 protein–protein interaction.^13^ Therefore, agents that interact with KEAP1 protein may liberate and activate the NRF2 pathway, which could further attenuate inflammatory acne.

Rhizoma Paridis is a traditional Chinese medicine, the most effective components of which are characterized as steroidal saponins. This traditional medicine has been used for long to treat inflammatory diseases and liver injury.^14^, ^15^ Over 60 steroidal saponins were isolated from Rhizoma Paridis, however, only a few of them have been well‐studied for their anti‐inflammatory activities and mechanisms.^15^, ^16^, ^17^ Polyphyllin I may inhibit heat‐killed *P*. *acnes* inducing IL‐6 and IL‐8 production in human keratinocytes by blocking the phosphorylation of P38 factor.^16^, ^17^ Polyphyllin VII was found to suppress LPS‐induced inflammation in RAW 264.7 cells and animal models.^18^ Although these studies showed the potential of polyphyllins to treat bacteria‐induced acne inflammation, the underlying mechanisms of these effects are far from well‐understood.

Our previous studies revealed that polyphyllin H (PPH) had similar effects with dexamethasone (DEX) in improving bacterial‐infected inflammatory skin equivalents.^19^ In this study, we aimed to further investigate the effects of PPH and PPH‐enriched Rhizoma Paridis extract (RPE) on Gram‐negative bacteria‐induced inflammatory acne by recruiting HaCaT and RAW 264.7 cell models. Moreover, the possible action mechanisms of PPH and other fractions of RPE were analysed through a combination of in silico analysis and molecular biology methodologies.

## 2 MATERIALS AND METHODS

### 2.1 Chemicals and reagents

PPH was purchased from Sarbio (SP5270, Beijing, China). RPE was prepared by Yunnan Baiyao Group Health Products Co., Ltd (Kunming, China). DEX (D4502, USA) and LPS (L2880, USA) were purchased from Sigma (Saint Louis, MO, USA). Abs against P38, JNK, ERK, AKT and phosphor‐P38 (p‐P38), p‐JNK, p‐ERK, p‐AKT were produced by Cell Signaling Technology (Danvers, MA, USA), those against NRF2, HO‐1, GAPDH were purchased from Proteintech (Rosemont, IL, USA), those against NF‐κB P65 and p‐P65 from Beyotime (Shanghai, China), and those against p‐NRF2 from Abcam (Waltham, MA, USA). Goat anti‐rabbit‐HRP and goat anti‐mouse secondary Abs were purchased from Bio‐Rad (Hercules, California, USA), while goat anti‐rabbit IgG (H&L) secondary Abs (DyLight 488) was purchased from Invitrogen (35552, Waltham MA, USA). ML385 was purchased form Selleck‐chem (S8790, USA) and RA839 was purchased from MedChemExpress (HY‐110275, USA). The Abs used in this study and related dilutions are reported in Table S1.

### 2.2 Cell culture and treatments

RAW 264.7 cells were seeded in six‐well plates at a concentration of 5 × 10^5^ cells/per well with 2 mL of Dulbecco's Modified Eagle Media (DMEM) high‐glucose medium (C11995500BT, Gibco‐Sigma, Saint Louis, MO, USA), and were cultured at 37°C with 5% CO_2_. For the LPS‐stimulated model group, RAW 264.7 cells were precultured for 6 h, then treated with 0.1 μg/mL of LPS for additional 24 h of incubation. For the LPS + DEX/RPE/PPH‐treated groups, RAW 264.7 cells were pretreated with DEX/RPE/PPH for 6 h before adding 0.1 μg/mL LPS. For the LPS + PPH + ML385‐treated groups, RAW 264.7 cells were treated with 0.125 μg/mL PPH and 5 μmol/L ML385 for 6 h before adding 0.1 μg/mL LPS. RAW 264.7 cells not treated with LPS and/or drugs were used as controls.

### 2.3 Total saponins determination

Total saponin quantification was performed as described by Baccou et al.^20^ with minor modification. RPE was dissolved in methanol to prepare solutions at the concentration of 0.2 mg/mL, and 1.5 mL RPE solution was air‐dried in a hot air bath. The pellet was mixed with 0.2 mL 8% reagent A (0.5% anisaldehyde, 99.5% ethyl acetate), then added 5 mL sulphuric acid. Samples were incubated in the 60°C water bath for 20 min, and quickly cooled down to room temperature (RT). Absorbance was measured at 450 mm. Commercial polyphyllin I was used to make a standard curve.

### 2.4 Cell viability measurement

The MTT cell proliferation and cytotoxicity assay kit (C0009, Beyotime, Shanghai, China) was used to assess the cell viability. Briefly, RAW 264.7 cells were cultured in DMEM high‐glucose medium using 96‐well plates with cells at a concentration of 1 × 10^4^ cells/per well and incubated overnight (37°C, 5% CO_2_). RAW 264.7 cells were treated with a gradient of concentrations for RPE or PPH for 24 h, and then washed twice with phosphate‐buffered saline (PBS) buffer (pH 7.4) (C10010500BT, Gibco‐Sigma, Saint Louis, MO, USA), while serum‐free DMEM was used in the control group cells. After 4 h of incubation, 10 μL of MTT (5 mg/mL) was added to each well, followed by formazan solubilization solution (100 μL). Absorbance at 570 nm was measured using a microplate reader (Tecan, Shanghai, China). The following formula was used to calculate the percentage of cell viability: OD_sample_/OD_blank_ × 100%.

### 2.5 Proinflammatory cytokine analysis

Cell culture supernatants were harvested for cytokine analysis. IL‐6 and IL‐1β contents were assessed by cytokine ELISA kit (KE10002 and KE10007, Proteintech, Rosemont, IL, USA) following the manufacturer's instruction.

### 2.6 Intracellular ROS analysis

RAW 264.7 cells at a concentration of 2.5 × 10^4^/per well were cultured in 24‐well plates overnight. Cells were treated with drugs (DEX/RPE/PPH) for 6 h, and followed by LPS treatment for 2 h, to finally collect RAW 264.7 cells to assess the ROS production by ROS assay kit (S0033S, Beyotime, Shanghai, China). An amount of 0.5 mL 10 mM DCFH‐DA was added to each well, following by an incubation at 37°C for 20 min. Cells were washed three times with serum‐free DMEM in order to remove the DCFH‐DA not penetrated into the cells. A 488‐nm excitation laser and a 525 nm emission filter were used to visualize the ROS signal on the microscope. The fluorescent intensities were quantified by ImageJ software (version 1.53a).

### 2.7 RNA extraction and qPCR analysis

Total RNA was extracted from RAW 264.7 cells using RNAios Plus reagent (Takara, Kusatsu, Japan) and RNA concentration was measured with a Nano‐300 ultramicro‐spectrophotometer (All Sheng, Zhejiang, China). TIANScript II RT Kit (KR107‐02, Tiangen, Shanghai, China) was used to synthesize cDNA. qPCR was performed using SYBR Green (11198ES08, YEASEN, Shanghai, China) on a Light Cycle® Real‐Time PCR machine (Roche, Switzerland). The primers used were synthesized by Sangon Biotech (Shanghai, China) (Table S2). *GAPDH* housekeeping gene was used as internal control. Transcript levels of target genes were calculated using the *2* ^−∆∆Ct^ method.

### 2.8 Western blot analysis

RAW 264.7 cells were treated with protein lysis buffer containing 1 mmol/L of PMSF (Beyotime, Shanghai, China). The total protein extract was obtained through centrifugation at 12,000 rpm for 10 min at 4°C. BCA protein assay kit (CW0014S, CWBiotech, Shanghai, China) was used to assess the protein concentration. The proteins (20 μg protein/lane) which were isolated by SDS–PAGE were transferred to a polyvinylidene fluoride membrane (Millipore‐Sigma, Burlington, MA, USA). Membranes were blocked in 5% skim milk at RT for 1 h, followed by an incubation with the primary antibody (Ab) overnight at 4°C. After washing three times with TBST (Tris‐buffered saline, 0.1% Tween 20), there was an incubation with the secondary Ab performed at RT for 2 h. The protein bands were captured using a Tanon 5200 multi chemiluminescence imaging system (TANON Science & Technology, Shanghai, China). Abs from this study are described in Table S1.

### 2.9 Immunofluorescence analysis

RAW 264.7 cells were cultured in chambered slides and then fixed in 4% paraformaldehyde for 10 min, following by PBS washing performed for three times. As for cell permeabilization treatments, cells were incubated in 1 mL of 0.01% Triton X‐100 for 10 min. After an additional PBS washing, cells were blocked with 10% normal goat serum at RT for 1 h. Cells were incubated with the primary Ab (diluted with 5% normal goat serum) overnight at 4°C followed by incubation with the goat anti‐rabbit IgG (H&L) secondary Ab (DyLight 488) at RT for 2 h. The cells mounted with hard‐set mounting medium with 4′, 6‐diamidino‐2‐phenylindole (DAPI) were then imaged using Nikon A1 confocal microscope (488 nm/525 nm, Japan). ImageJ software (version 1.53a) was used for the quantification of the fluorescence intensity. The nucleus regions were selected by the wand tool in ImageJ. % Nuclear intensity of NF‐κB P65 was calculated as: the fluorescence intensity of NF‐κB P65 within the nucleus/the total fluorescence intensity of NF‐κB P65.

### 2.10 Molecular docking

The structure of PPH, polyphyllin VI and polyphyllin VII were drawn by using ChemDraw Professional, and was then converted into Mol2 format files by Chem3D 17.0. To optimize the molecular structures and obtain energetically favourable conformations, energy minimization calculations were performed using the Tripos force field. This conformation process was carried out using SYBYL‐X 2.0. The crystal structure of KEAP1 (PDB code: 4l7b) was obtained from the PDB database (http://www.pdb.org). Prior to the docking procedure, all water molecules and co‐crystallized ligands were removed from the KEAP1 structure. Subsequently, hydrogen atoms were added and gasteiger charges were computed. The protein structure was saved as Mol2 format files. KEAP1 and corresponding small molecules were then docked in SYBYL‐X 2.0 by using Surflex‐Dock docking mode. The binding energy was calculated and the molecular interaction was visualized by Pymol 2.4.0 visualization tool.

### 2.11 Surface plasmon resonance (SPR) analysis

SPR analysis was performed following Jiang et al.^21^ In brief, human KEAP1 protein was immobilized on a CM5 chip (Cytiva, USA). The analysis was carried out on a Biacore TM T200 instrument (GE Healthcare, USA). PPH was diluted with the running buffer (PBS with 5% DMSO) to a range of concentrations (1.80, 3.59, 7.19, 14.38, 28.75, 57.5, 115 and 230 μM). PPH was injected at a flow rate of 30 μL/min followed by a 60‐s contact phase and 120‐s dissociation phase. The affinity value (equilibrium dissociation constant, *K*_*D*_) was calculated by using Biacore T200 Evaluation Software.

### 2.12 Statistical analysis

GraphPad PRISM 8 software (GraphPad Software, Boston, USA) was used for all statistical analyses performed in this study employing a Student's *t*‐test.

## 3 RESULTS

### 3.1 PPH reduced Gram‐negative bacterial PAMP‐induced intracellular ROS generation

**FIGURE 1:**
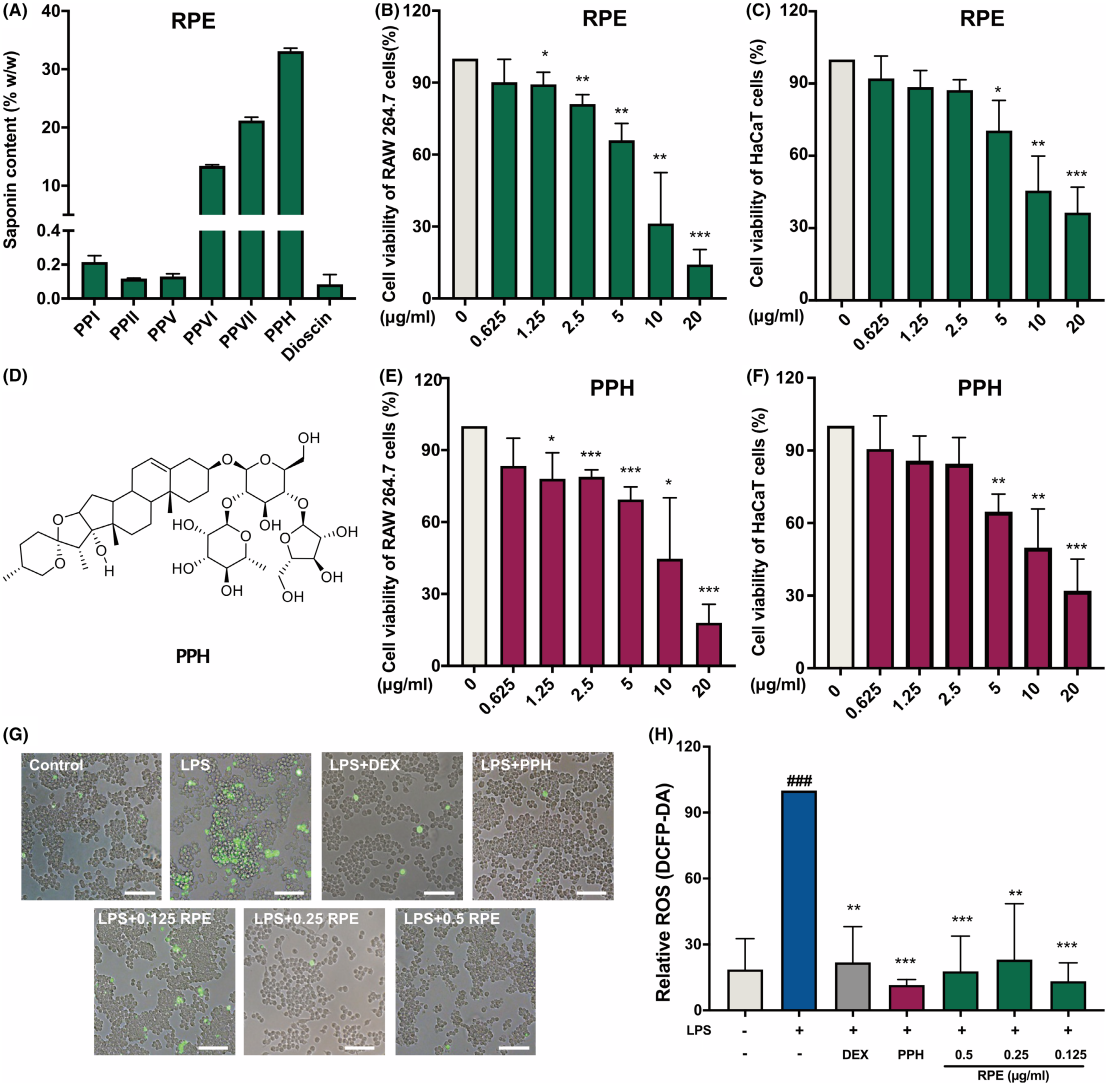
Effects of RPE and PPH on cell viability and intracellular ROS production. The saponin content of RPE The polyphyllin (PP) profiling of RPE assessed by HPLC (A). The chemical structure of PPH (D). The effects of gradient concentrations of PRE (B) and PPH (E) on the viability of RAW 264.7 cells. The effects of gradient concentrations of PRE (C) and PPH (F) on the viability of HaCaT cells. Data were collected from three independent experiments. *p < 0.05, **p < 0.01, ***p < 0.001 (Student's *t*‐test, treatment vs. blank control). (G) Fluorescence images of intracellular ROS generations in LPS‐stimulated RAW 264.7 cells. Bar = 100 μm. Fluorescence intensities of intracellular ROS assessed by ImageJ (H). Data were collected from three independent experiments. *p < 0.05, **p < 0.01, ***p < 0.001 (Student's *t*‐test, treatment vs. LPS‐induced group). ^###^ *p* < 0.001 (Student's *t*‐test, LPS‐induced group vs. blank control). Error bars show the standard deviation.

To investigate the potential bioactive components of RPE, the total saponin content and polyphyllin profiling were first analysed. The total saponin content of RPE was 71.49 ± 3.56%, while high‐performance liquid chromatography (HPLC) revealed that PPH, polyphyllin VII and polyphyllin VI were the most abundant, accounting the 33.1%, 21.2%, and 13.4% (w/w), of RPE, respectively (Figure 1A and Figure S3). Polyphyllin I, II, V and dioscin were found with very low abundance (lower than 0.5%). To investigate the possible role of the most enriched polyphyllin in RPE, a commercial PPH‐treated group was include in the subsequent experiments (Figure 1D), and the cytotoxicity of PPH and RPE on HaCaT and RAW 264.7 cells were evaluated by the 3‐[4,5‐dimethylthiazol‐2‐yl]‐2,5 diphenyl tetrazolium bromide (MTT) assay (Figure 1 and Figure S1). RPE and PPH treatment were found to decrease cell viability in a dose‐dependent manner (Figure 1B,C,E,F). Up to 0.625 μg/mL of PPH and RPE showing no significant cytotoxicity on HaCaT and RAW 264.7 cells. Given the possible cytotoxicity effect of LPS on skin cells, the cell viability of RPE and PPH were analysed in LPS‐induced cell models as well (Figures S1 and S2). At a concentration of 0.1 μg/mL of LPS, we found that the cell viability in PPH‐ and RPE‐treated cells were 70.7% and 72.5%, respectively (Figure S1). In the following experiments, the RPE with no cytotoxic effect (0.125, 0.25 and 0.5 μg/mL) was used. The concentration of commercial PPH monomer (0.125 μg/mL) used in this study was consistent with the concentration of PPH in 0.5 μg/mL of RPE was selected. DEX, being a typical drug for chronic condition treatment, was used as a positive control. 2′,7′‐dichlorofluorescein diacetate (DCFH‐DA) assay was performed to evaluate the ROS scavenging activity in the different groups (Figure 1G,H). Results showed that LPS treatment dramatically stimulated the intracellular ROS generation, whereas preincubation with DEX, PPH and RPE significantly inhibited the over‐accumulation of ROS induced by LPS. Together, these findings suggested that PPH and RPE might prevent the oxidative stress induced by Gram‐negative bacterial PAMP.

### 3.2 PPH and RPE attenuated LPS‐induced proinflammatory cytokines expressions both in keratinocytes and macrophages

**FIGURE 2:**
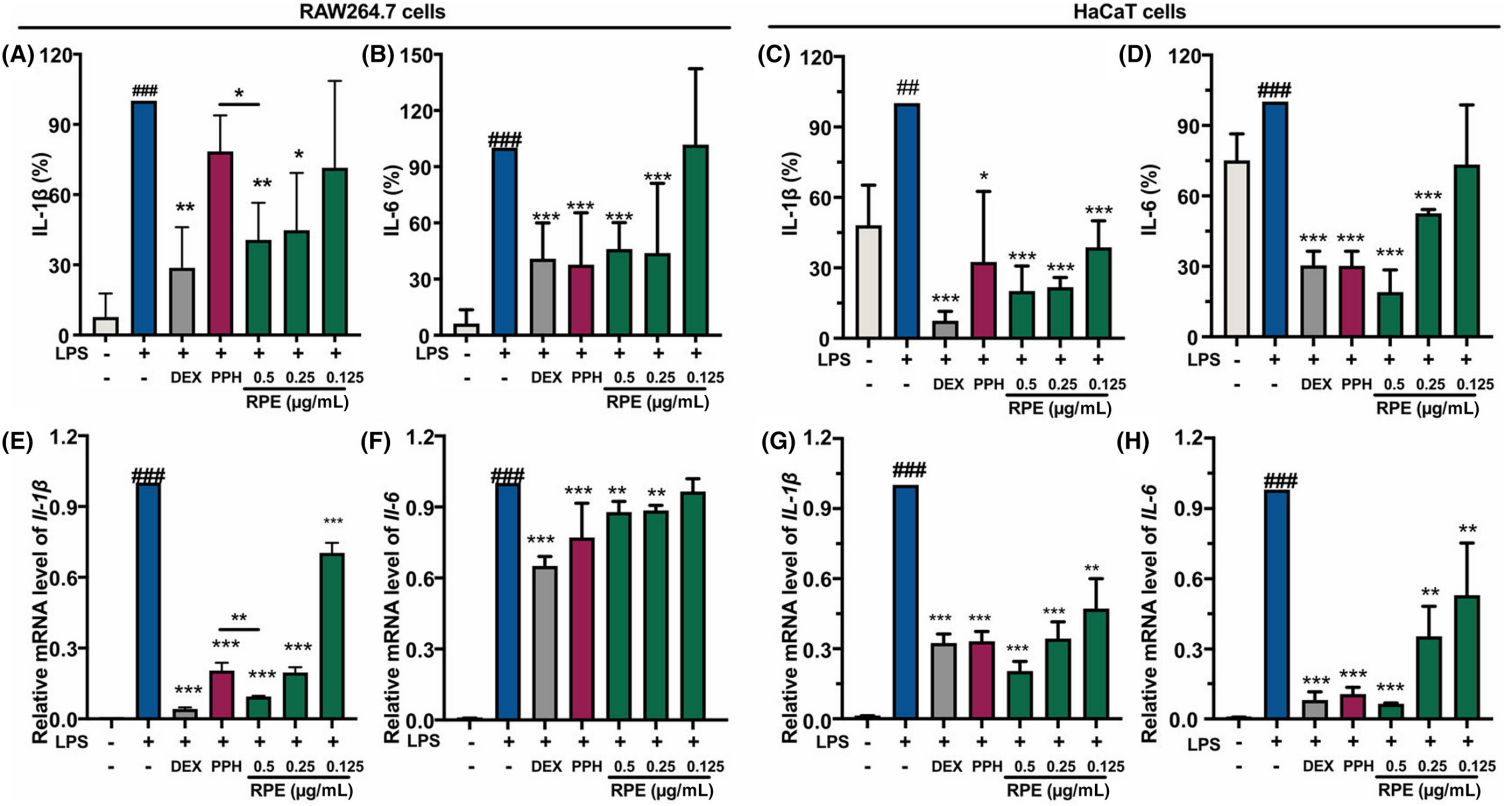
RPE and its main component PPH inhibited the expression of proinflammatory factors in LPS‐induced RAW 264.7 cells and HaCaT cells. The effects of RPE and PPH on the production of IL‐1β and IL‐6 in LPS‐induced RAW 264.7 cells (A) and (B). The effects of RPE and PPH on the production of IL‐1β and IL‐6 in LPS‐induced HaCaT cells (C) and (D). The effects of RPE and PPH on the mRNA expression of *Il‐1β* and *Il‐6* in LPS‐induced RAW 264.7 cells (E) and (F). The effects of RPE and PPH on the mRNA expression of interleukin *IL‐1β* and *IL‐6* in LPS‐induced HaCaT cells (G) and (H). RAW 264.7 cells were pretreated with 50 μg/mL dexamethasone (DEX), 0.125 μg/mL PPH, and different concentrations of RPE (0.125, 0.25, and 0.5 μg/mL), respectively. *Gapdh* and *GAPDH* were used as internal controls in RAW 264.7 cells and HaCaT cells, respectively. *p < 0.05, **p < 0.01, ***p < 0.001 (Student's *t*‐test, treatment vs. LPS‐induced group). ^###^ *p* < 0.001 (Student's *t*‐test, LPS‐induced group vs. blank control). Data were collected from three independent experiments. Error bars show standard deviation.

We next investigated the effects of RPE and PPH on the induction of key proinflammatory cytokines during the acne pathogenesis (IL‐1β and IL‐6) in LPS‐stimulated RAW 264.7 cells (Figure 2A,B,E,F). Compared with the control group, the IL‐1β and IL‐6 levels in LPS‐stimulated RAW 264.7 cells were significantly increased (Figure 2A,B), while RPE treatment decreased their secretions in a dose‐dependent manner. In addition, the mRNA expression of *Il‐1β* and *Il‐6* were also analysed by qPCR (Figure 2E,F), and similarly, RPE was found able to inhibit their mRNA expressions induced by LPS in a concentration‐dependent way. No significant differences were found between the 0.5 μg/mL RPE‐ and DEX‐treated groups. However, compared with PPH‐treated RAW 264.7 cells, 0.5 μg/mL RPE treatment resulted in a greater inhibition of the suppression of protein and mRNA expression of IL‐1β. The anti‐inflammatory activities of RPE and PPH were also evaluated in LPS‐induced HaCaT cells (Figure 2C,D,G,H). Results revealed that RPE and PPH suppressed the mRNA expression and secretion of IL‐6 and IL‐1β induced by LPS. Together, RPE and PPH downregulated the expressions of proinflammatory cytokines in LPS‐induced RAW 264.7 and HaCaT cells, which suggested that RPE and PPH may attenuate the Gram‐negative bacteria‐induced inflammation.

### 3.3 RPE and PPH suppressed NF‐κB P65 nuclear translocation in LPS‐stimulated RAW 264.7 cells

**FIGURE 3:**
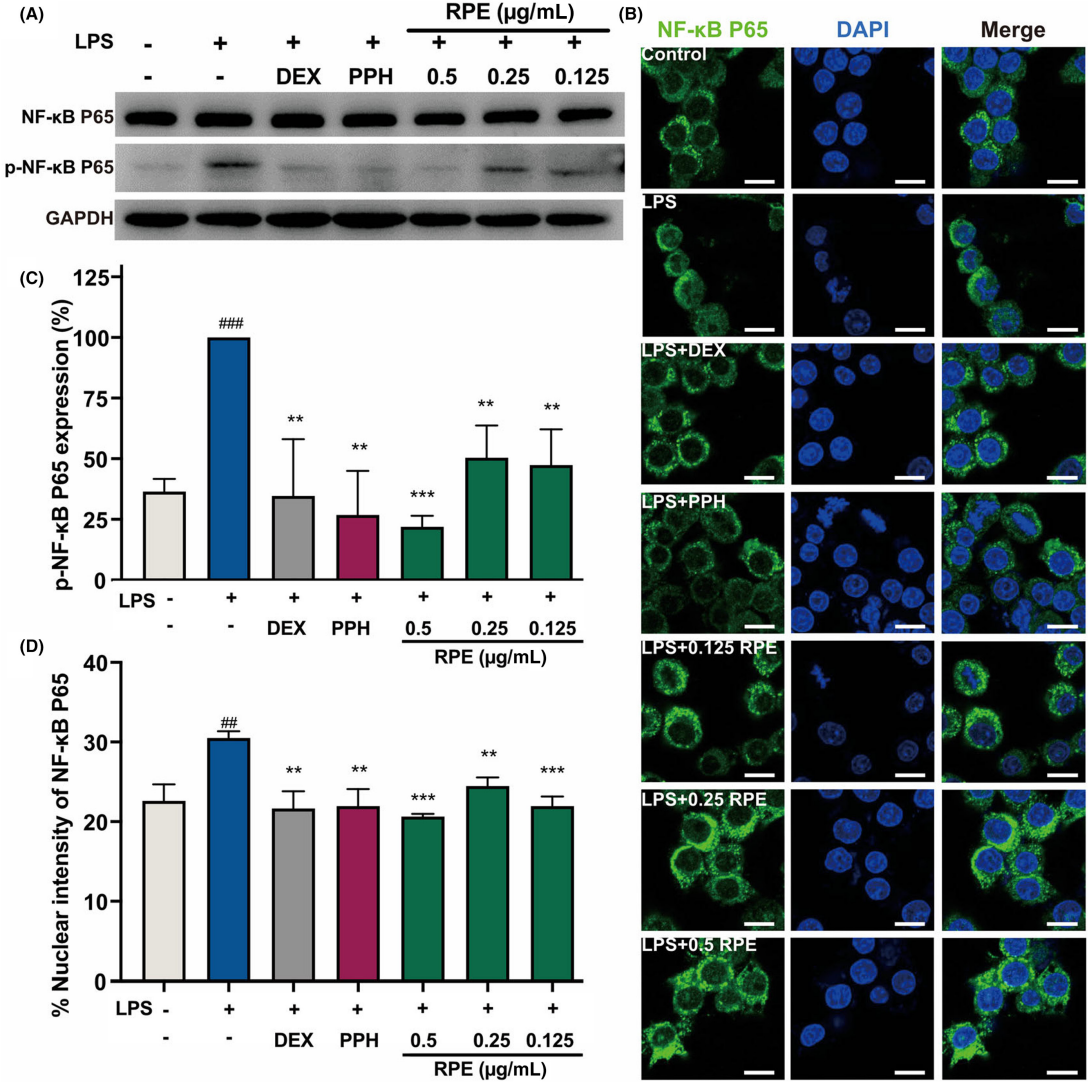
RPE and its main component PPH blocked the nuclear translocation of NF‐κB P65 in LPS‐induced RAW 264.7 cells. Effects of RPE and PPH on the expression of NF‐κB P65 and p‐NF‐κB P65 induced by 0.1 μg/mL of LPS in RAW 264.7 cells (A). Immunofluorescence staining of NF‐κB P65 in fixed RAW 264.7 cells treated with/without LPS for 24 h. The nuclear regions were visualized by DAPI. Bar = 10 μm (B). Expression levels of p‐NF‐κB P65 quantified by WB using ImageJ software (C). Immunofluorescence quantification of NF‐κB P65 at the nuclear regions. RAW 264.7 cells were pretreated with 50 μg/mL dexamethasone (DEX), 0.125 μg/mL PPH, and different concentrations of RPE (0.125, 0.25, and 0.5 μg/mL), respectively (D). **p < 0.01, ***p < 0.001 (Student's *t*‐test, treatment vs. LPS‐induced group). ##*p* < 0.01, ^###^ *p* < 0.001 (Student's *t*‐test, LPS‐induced group vs. blank control). GAPDH was used as the internal control. Data was collected from three independent experiments. Error bars show standard deviation.

The inflammation regulation mechanisms of PPH and RPE were further investigated. Since NF‐κB interacts with ROS and induces proinflammatory gene expressions, we determined the protein expressions and phosphorylation levels of NF‐κB P65 protein in RAW 264.7 cells by WB (Figure 3A,C). LPS induced a statistically significant increase of the phosphorylation levels of NF‐κB P65 without altering its protein expressions. On the other hand, DEX, RPE and PPH treatments significantly decreased the phosphorylation levels of NF‐κB P65 (Figure 3C). Moreover, an immunofluorescence staining was performed to detect the nuclear translocation status of NF‐κB P65 (Figure 3B). In the LPS‐stimulated group, a 1.5‐fold increase in the abundance of NF‐κB P65 protein was observed in the nuclear regions (Figure 3D). However, PPH, RPE and DEX treatment resulted in a decreased signals of activated NF‐κB P65 in the nuclear region. Therefore, these findings supported that PPH can inhibit the expression of NF‐κB P65 protein, resulting in less expressions of proinflammatory cytokines and intracellular ROS production.

### 3.4 PPH was selectively bind to KEAP1 protein

**FIGURE 4:**
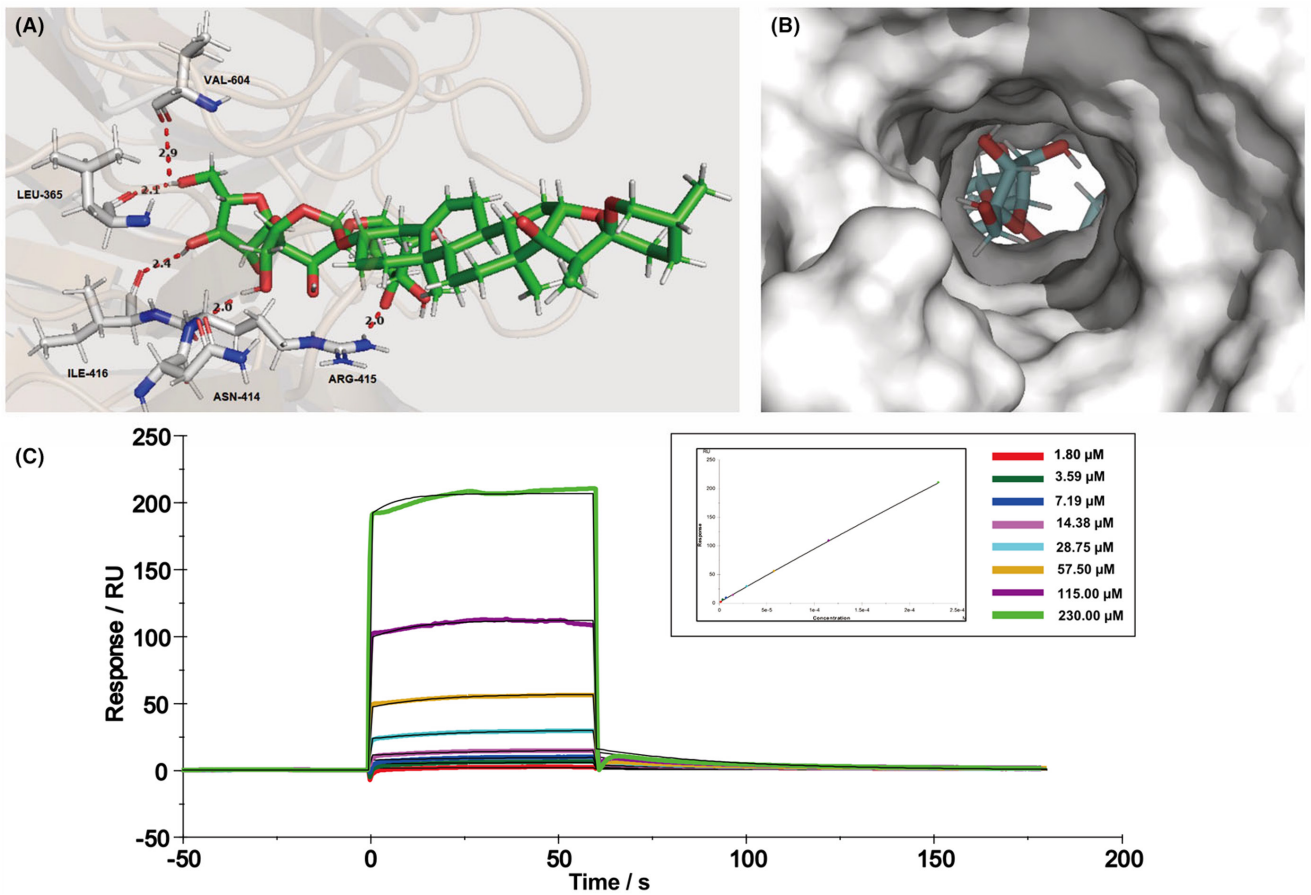
Interaction between PPH and KEAP1 protein. Docking model of PPH with KEAP1 (A,B). PPH forms five hydrogen bonds with KEAP1 (A). PPH interacts with the binding pocket of KEAP1 (B) (PDB ID: 4l7b). Surface Plasmon Resonance (SPR) Analysis of PPH‐KEAP1 Interaction (C).

Given that PPH and RPE had inhibitory effects on ROS production (Figure 1G,H), we hypothesized that polyphyllins present in RPE might play a role in the KEAP1/NRF2 signalling pathway. To investigate this possibility, the molecular docking was performed between KEAP1 protein and the three most abundant polyphyllins of RPE (PPH, polyphyllin VI and polyphyllin VII), respectively. It was found that only PPH showed high binding affinity with KEAP1 protein by forming five hydrogen bonds within the binding pocket region of KEAP1 (Figure 4A,B, and Table S3). In addition, the hydrogen atoms of hydroxyl groups at tetrahydrofuran were able to form hydrogen bonds with the VAL‐604, LEU‐365 and ILE‐416 residues with the corresponding distances of 2.9 Å, 2.1 Å and 2.4 Å, respectively. The hydroxy hydrogen atoms of tetrahydropyran also were involved in the noncovalent interactions with ASN‐414 and ARG‐415 residues through hydrogen bond reciprocity with a same space distance of 2.0 Å. Furthermore, we performed SPR analysis to assess the binding interaction between PPH and KEAP1 protein. The binding affinity of PPH with KEAP1 showed a dose‐dependent manner, with a *K*_*D*_ of 3.877 mM (Figure 4C). Overall, these results provided the in silico and experimental evidence that PPH is able to bind KEAP1 protein.

### 3.5 PPH activated the NRF2/HO‐1 antioxidant signalling pathway

**FIGURE 5:**
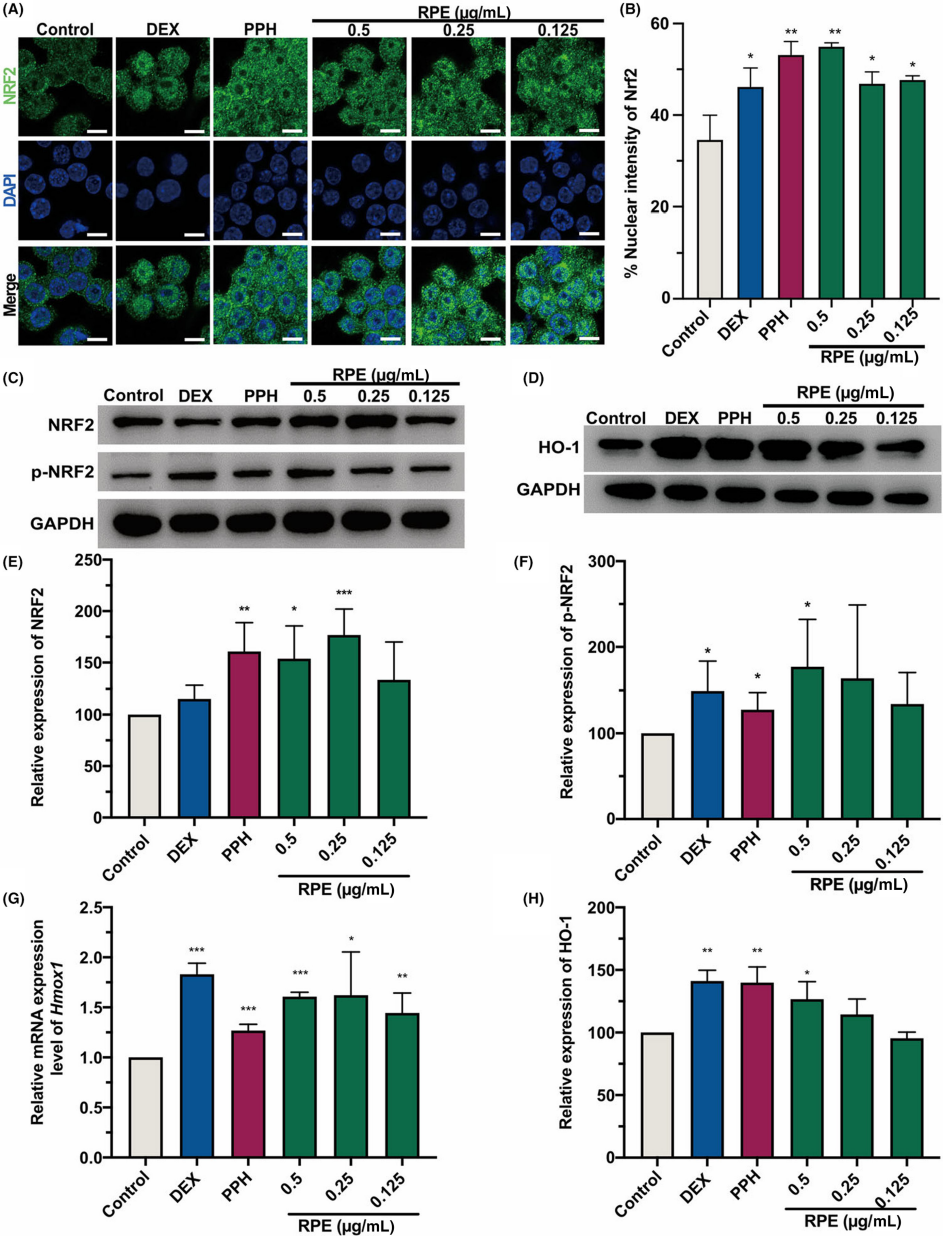
PPH activated the NRF2/HO‐1 signalling pathway. Immunofluorescence staining of NRF2 in fixed RAW 264.7 cells treated with PPH, RPE, and DEX, respectively, for 6 h. The nuclear regions were stained with DAPI. Bar = 10 μm (A). Relative abundance of NRF2 in nuclear regions quantified by using ImageJ software (B). Effects of PPH and RPE on the expressions of NRF2 and p‐NRF2 (C) and HO‐1 (D) were analysed by WB. Relative expression levels of NRF2 (E), p‐NRF2 (F), and HO‐1 (H) were quantified by ImageJ software. (G) The effects of RPE and PPH on the mRNA expression of *Hmox1*. *Gapdh* housekeeping gene was used as internal control. RAW 264.7 cells were treated with 50 μg/mL of DEX, 0.125 μg/mL PPH, and different concentrations of RPE (0.125, 0.25, and 0.5 μg/mL), respectively. *p < 0.05, **p < 0.01, ***p < 0.001 (Student's *t*‐test, treatment vs. blank control). Data were collected from three independent experiments. Error bars show standard deviation.

To assess the potential role of PPH as an activator of the NRF2 signalling pathway, we assessed its expression in RAW 264.7 cells treated with PPH, RPE and DEX (Figure 5C,E,F). WB results showed that PPH and RPE both increased the protein and phosphorylation levels of NRF2, whereas DEX only significantly activate NRF2 (Figure 5C,E,F). In addition, an immunofluorescence analysis was performed to detect NRF2 activation in the nucleus of RAW 264.7 cells (Figure 5A,B). As shown in Figure 5B, PPH and RPE significantly enhanced the fluorescence intensity of NRF2 in the nuclear regions of RAW 264.7 cells. The role of PPH in upregulating the protein and phosphorylation levels of NRF2 in RAW 264.7 cells was confirmed by quantifying the fluorescence intensity of NRF2. In addition, we detected the expression of the downstream factor, HO‐1 (Figure 5G,H). The mRNA and protein expression of HO‐1 were both increased in the PPH and RPE treatment groups, along with the DEX‐treated group.

To confirm the regulation of intracellular ROS generation by PPH via the NRF2/HO‐1 pathway, we recruited ML385, a widely used NRF2 inhibitor with reported concentration for our further analysis.^22^, ^23^ Immunolabelling results revealed that ML385 treatment inhibited the PPH‐stimulated activation of Nrf2, as evidenced by reduced fluorescence intensity in the nuclear region (Figure S4). In addition, the increased mRNA expression of *Hmox1* induced by PPH treatment was attenuated in the ML385‐treated group (Figure S5). Consistently, ML385 impaired the inhibitory effect of PPH on LPS‐induced ROS production (Figure S6). Together, these results supported our hypothesis that PPH can attenuate the inflammatory response and oxidative stress by modulating the NRF2/HO‐1 signalling pathway.

### 3.6 RPE might also modulate NF‐κB activation by suppressing the activation of P38 and JNK

**FIGURE 6:**
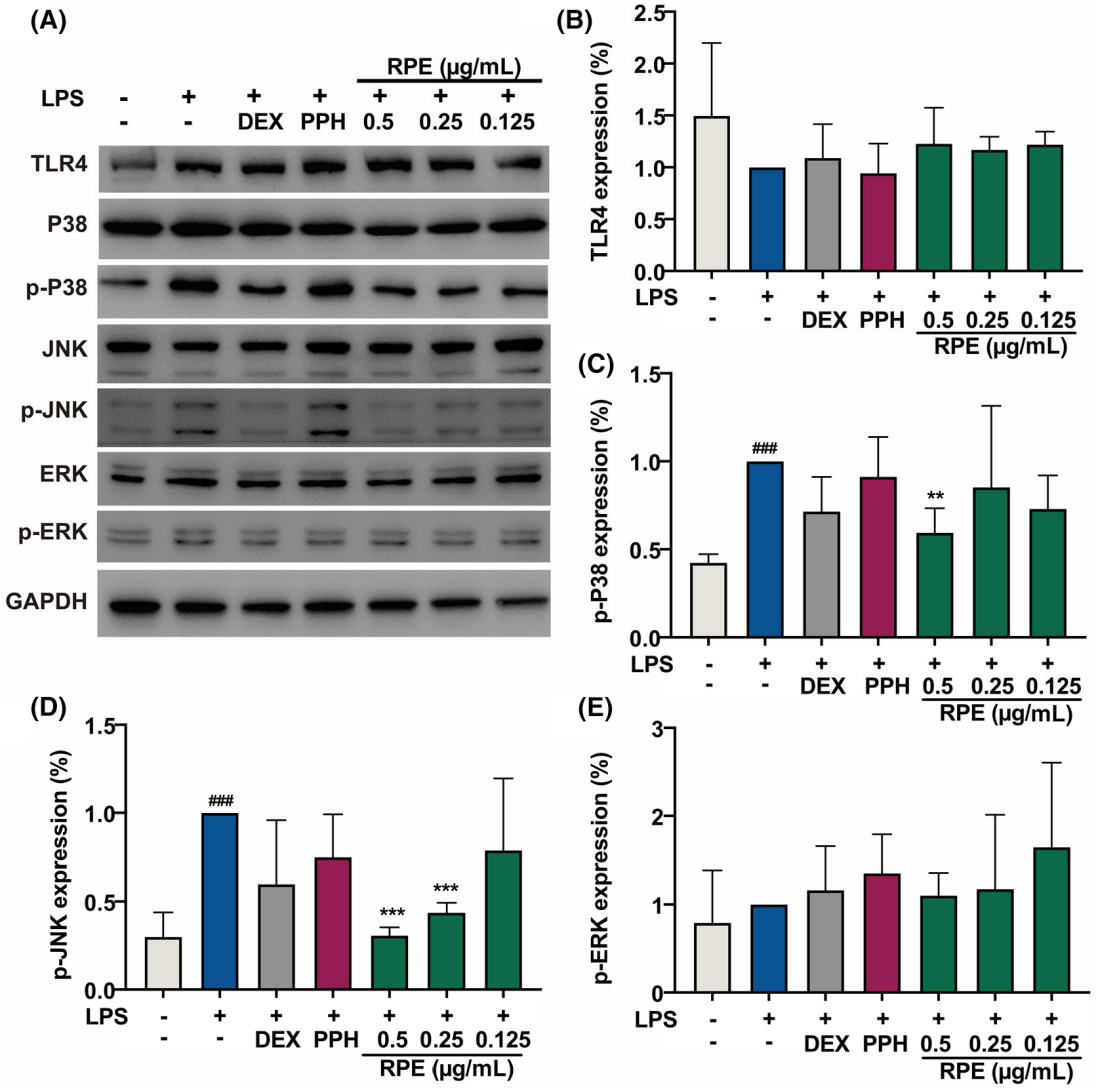
RPE modulated the MAPK signalling pathway. WB analysis of the effects of RPE and PPH on the expression of TLR4 and key factors in the MAPK pathway in LPS‐induced RAW 264.7 cells (A). Expression levels of TLR4 (B), p‐P38 (C), p‐JNK (D), and p‐ERK (E) were quantified by WB using ImageJ software. RAW 264.7 cells were pretreated with 50 μg/mL of DEX, 0.125 μg/mL PPH, and different concentrations of RPE (0.125, 0.25, and 0.5 μg/mL), respectively. **p < 0.001, ***p < 0.001 (Student's *t*‐test, treatment vs. LPS‐induced group). ^###^ *p* < 0.001 (Student's *t*‐test, LPS‐induced group vs. blank control). Data were collected from three independent experiments. Error bars show standard deviation.

To understand whether RPE and PPH could modulate the NF‐κB activation through the regulation of upstream pathways, we also analysed the expression and activation of TLR4 and key MAPK components in LPS‐stimulated RAW 264.7 cells (Figure 6). Western results showed that neither LPS induction nor drug treatment affected the protein expressions of TLR4, P38, JNK and ERK (Figure 6A,B). In the LPS‐induced group, phosphorylation levels of P38, JNK and ERK were found to be significantly increased (Figure 6C–6E), while the RPE treatment significantly reduced the phosphorylation level of JNK in a dose‐dependent manner (Figure 6D). In addition, RPE at a concentration of 0.5 μg/mL also suppressed the activation of P38, whereas the phosphorylation level of ERK was not altered (Figure 6C,E). Surprisingly, phosphorylation levels of P38, JNK and ERK in the PPH‐treated group did not report differences with the LPS‐stimulated group, indicating a lack of role of PPH in the MAPK signalling pathway. The different roles of RPE and PPH on the MAPK signalling pathway indicated that the non‐PPH components of RPE might inhibit inflammation through the suppression of JNK and P38 activation.

## 4 DISCUSSION

Although the complex pathogenesis of acne has not yet been fully understood, the essential role of the inflammation in the development of acne was ascertained. The most recent researches have advanced our understanding that Gram‐negative bacteria are key inflammation‐inducers in acne development.^8^, ^9^ However, only a few antiacne agents which may act also against Gram‐negative were reported. In this study, we demonstrated the anti‐inflammatory activity of Rhizoma Paridis saponins, and elucidated the specific underlying mechanisms used by different Rhizoma Paridis saponins in treating bacteria‐induced inflammatory acne.

Acne can be classified into Grade I–Grade IV. In details, it is characterized by comedones (Grade I); inflammatory papules (Grade II); papules and pustules (Grade III); and nodules/cysts (Grade IV).^2^ Keratinocytes, macrophages and sebocytes are all involved in the development of acne.^6^ Specifically, the accumulation of keratinocytes in the ductal infundibulum causes hyperkeratosis and induces proinflammatory cytokines, including IL‐1, IL‐6 and TNF‐α, resulting in a closed or open comedones (Grade I). The dysfunction of sebocytes can lead to excessive sebum production and upregulated inflammatory mediators. Additionally, macrophages also upregulate the immune responses by increasing the cytokines, further resulting in the severe inflammation typical of acne of Grade III–Grade IV. The IL‐1β and IL‐6 released by macrophages are the main triggering factors of innate immune response. Keratinocyte cell lines, especially HaCaT, are commonly used to evaluate their antiacne activities.^16^, ^17^ To investigate agents that target multiple pathological processes of acne, we used both keratinocytes and macrophages in this study. We demonstrated that PPH and RPE significantly reduced the expressions of IL‐1β and IL‐6 in keratinocyte line, HaCaT and macrophage line, RAW 264.7 (Figure 2). In addition, RPE showed a dose‐dependent inhibitory effect on LPS‐induced inflammation, suggesting that PPH might control the development of early comedones and suppress infiltration of activated immune cells as well.

As the typical component of Gram‐negative bacterial cell wall, LPS triggers the pattern recognition response of skin cells and activates the innate immune response.^10^ Therefore, LPS‐induced inflammation models were used in study. Consistently with previous studies, LPS significantly induced the mRNA and protein expression of inflammatory cytokines and ROS generation in HaCaT and RAW 264.7 cells (Figures 1G,H and Figure 2). In addition, the signals of NF‐κB P65 within the nucleus were increased in LPS‐stimulated macrophages, leading to an enhanced transcription of inflammatory cytokines. Our immunofluorescence staining results demonstrated that PPH and RPE treatment inhibited the nuclear translocation of the LPS‐stimulated NF‐κB P65, consistently with the decreased phosphorylation level of NF‐κB P65 in PPH‐ and RPE‐treated macrophages (Figure 3). Together, these data indicated that PPH and RPE may regulate the LPS‐induced inflammation through the NF‐κB‐dependent pathway.

Intracellular ROS could induce the activation of NF‐κB, and therefore, the cellular antioxidant pathways are essential for regulating inflammatory responses.^12^ Indeed, the activation of the NRF2/HO‐1 pathway may upregulate the expression of antioxidant enzymes, which further downregulates the oxidative stress. Moreover, the upregulated HO‐1 is able to inhibit the transcription regulation of NF‐κB, resulting in a reduced expression of downstream inflammatory mediators. A more recent study reported that NRF2 decreased the LPS‐induced expression of IL‐6 and IL‐1β in macrophages by binding to the protein‐encoding genes.^11^ Plant‐derived coumarins compounds were reported as antioxidant and anti‐inflammatory agents that could interact with KEAP1 protein.^13^ We provided in silico and SPR evidence that PPH could bind to the KEAP1 protein (Figure 4), whereas a low binding affinity was found for other major polyphyllins of RPE. In this study, we also confirmed the role of PPH in the upregulation of the NRF2/HO‐1 pathway. Consistently, a significantly increased NRF2 signal was found within the nucleus of PPH‐treated RAW 264.7 cells (Figure 5). Surprisingly, we found that PPH could induce both the protein expression and activation of NRF2, whereas DEX acts only on the phosphorylation level of NRF2. These findings might explain the higher nuclear intensity of NRF2 in the PPH‐treated group than in the group treated with DEX. Besides PPH, the coumarins compound umbelliferone was also reported able to increase the mRNA expression of *Nrf2* in the rat model.^13^, ^24^ We found indeed that the *Hmox1* mRNA and HO‐1 protein were significantly upregulated in PPH‐treated macrophages, confirming that PPH can activate the NRF2/HO‐1 pathway through binding to the KEAP1 protein. These results indicate the peculiar role of PPH in the regulation of inflammation.

Polyphyllins are a major group of active components of RPE,^15^ and different polyphllins were found to have distinct effects on the regulation of the acne inflammation.^16^, ^17^ Our previous study found that PPH could inhibit the production of proinflammatory cytokines induced by *P. acnes* in vitro,^19^ while PPVII was found able to inhibit the mRNA and protein expression of IL‐1β and IL‐6 in LPS‐induced RAW 264.7 cells and animal models.^18^ Additionally, PPI attenuates *P. acnes* induced inflammation decreasing IL‐6, IL‐8 and TNF‐α in HaCaT cells.^16^, ^17^ The effects of PPH and RPE at the same dose of PPH were object of a comparative analysis in this study (Figure 2). The anti‐inflammatory activities of other polyphyllins of RPE explains the reason for which RPE has higher activities than PPH. Indeed, our studies for the first time demonstrated that PPH is a polyphyllin with anti‐inflammatory activities against both Gram‐negative and Gram‐positive bacteria‐induced cell models. Moreover, we unveiled that PPH specifically activates the antioxidative pathway through the binding to KEAP1, further determining the NRF2 accumulation. On the contrary, non‐PPH components of RPE affected the MAPK signalling pathway through the inhibition of JNK and activation of P38. It has been reported that PPVII could inhibit the activation of ERK, P38 and JNK in LPS‐induced RAW 264.7 cells.^18^ Since PPVII is enriched in RPE, it is possible that PPVII plays a role in the anti‐inflammatory activity of the non‐PPH fractions.

In summary, we demonstrated that PPH and PPH enriched extract‐RPE has a high anti‐inflammatory and antioxidant activity in LPS‐induced macrophages and keratinocytes, determining a reduction in the proinflammatory cytokine expressions and intracellular ROS generation. Additionally, PPH could upregulate the NRF2/HO‐1 antioxidant pathway by binding to the KEAP1 protein, while non‐PPH fractions of RPE could inhibit the activation of the MAPK pathway. Both processes together suppress the nuclear translocation of NF‐κB and downstream inflammatory genes expression. Given that PPH also inhibits *P. acnes*‐induced inflammation, PPH‐enriched RPE may represent a promising agent for the treatment of both Gram‐negative and Gram‐positive bacteria‐induced acne.

## AUTHOR CONTRIBUTIONS

**Yang Yang:** Conceptualization (equal); formal analysis (equal); writing – original draft (equal). **Chaofan Wang:** Investigation (equal); writing – original draft (equal). **Juan Wang:** Investigation (equal). **Lingli Yang:** Investigation (equal). **Zheng Lv:** Investigation (equal). **Quan An:** Conceptualization (equal). **Yiming Wang:** Formal analysis (equal). **Xue Shao:** Investigation (equal). **Fei Wang:** Formal analysis (equal). **Tong Huo:** Formal analysis (equal). **Jiali Liu:** Conceptualization (equal); writing – review and editing (equal). **Haoshu Luo:** Conceptualization (equal); writing – review and editing (equal). **Qianghua Quan:** Conceptualization (equal); writing – review and editing (equal).

## FUNDING INFORMATION

This research was funded by Yunnan Science and technology project (grant number 2018ZF005).

## CONFLICT OF INTEREST STATEMENT

Yang Yang, Lingli Yang, Yiming Wang, Xue Shao, Fei Wang, Tong Huo, and Quan An are employed by Yunnan Baiyao Group Health Products Co., Ltd. Juan Wangs was employed by Yunnan Baiyao Group Health Products Co., Ltd.

## Supporting information


Appendix S1:

## Data Availability

All data generated during this study are included in this article and its supplementary information files.
