# Supplementary material for: Rhizoma Paridis saponins attenuate Gram‐negative bacteria‐induced inflammatory acne by binding to KEAP1 and modulating Nrf2 and MAPK pathways
Source: J Cell Mol Med. 2024 Mar 1;28(6):e18146. doi: 10.1111/jcmm.18146 (PMC10906378; doi:10.1111/jcmm.18146)
Supplement: Supplementary file 1 — Appendix S1: [file JCMM-28-e18146-s001.docx]

**Supplementary Table 1.** Antibodies used in this study

| ANTIBODIES | SOURCE | IDENTIFIER |
| --- | --- | --- |
| Rabbit anti-TLR4 (WB, 1:2000) | Proteintech | 66350-1-Ig |
| Rabbit anti-P38(WB, 1:1000) | Cell Signaling Technology | [8690](http://www.ab-mart.com.cn/page.aspx?node=%2065%20&id=%2050922) |
| Rabbit anti-p-P38(WB, 1:1000) | Cell Signaling Technology | 4511 |
| Rabbit anti-JNK (WB, 1:1000) | Cell Signaling Technology | 9252 |
| Rabbit anti-p-JNK (WB, 1:1000) | Cell Signaling Technology | 4668 |
| Rabbit anti-ERK (WB, 1:1000) | Cell Signaling Technology | 4695 |
| Rabbit anti-p-ERK (WB, 1:1000) | Cell Signaling Technology | 4370 |
| Rabbit anti-AKT(WB, 1:1000) | Cell Signaling Technology | 4691s |
| Rabbit anti-p-AKT(WB, 1:1000) | Cell Signaling Technology | 9275s |
| Rabbit anti-P65(WB, 1:1000 IF,1:100) | Beyotime | AF1234 |
| Rabbit anti-p-P65(WB, 1:1000) | Beyotime | AF5881 |
| Rabbit anti-NRF2 (WB, 1:3000 IF,1:300) | Proteintech | 16396-1-AP |
| Rabbit anti-p-NRF2 (WB, 1:5000) | Abcam | Ab76026 |
| Rabbit anti-HO-1 (WB, 1:3000) | Proteintech | 10701-1-AP |
| Mouse anti-GAPDH (WB,1:10000) | Proteintech | 60004-1-Ig |
| GAR-488 (IF, 1:800) | invitrogen | 35552 |
| GAR-HRP (WB,1:8000) | Bio-Rad | #1705046 |
| GAM-HRP (WB,1:8000) | Bio-Rad | #1705047 |

**Supplementary Table 2.** Primers used in this study

| **Target** | **Sequence (5' to 3')** |
| --- | --- |
| *Hmox1* (M)-F | CTTCCCGAACATCGACAGCC |
| *Hmox1* (M) -R | CAGCTCCTCAAACAGCTCAAT |
| *Il-6* (M)-F | GACTGGGGATGTCTGTAGCTC |
| *Il-6* (M)-R | CAACTGGATGGAAGTCTCTTGC |
| *Il-1β* (M)-F | TGCCACCTTTTGACAGTGATG |
| *Il-1β* (M)-R | ATGTGCTGCTGCGAGATTTG |
| *Gapdh* (M)-F | ТССТССАССАССААСТССТТАG |
| *Gapdh* (M)-R | ATGACCTTGCCCACAGCCTTG |
| *IL-6* (H)-F | ACATCCTCGACGGCATCTCA |
| *IL-6* (H)-R | CACCAGGCAAGTCTCCTCATT |
| *IL-1β* (H)-F | AAATGATGGCTTATTACAGTGGCA |
| *IL-1β* (H)-R | CCCTTGCTGTAGTGGTGGTC |
| *GAPDH* (H)-F | GGAGCGAGATCCCTCCAAAAT |
| *GAPDH* (H)-R | GGCTGTTGTCATACTTCTCATGG |

**Supplementary Table 3**. Molecular docking scores of polyphyllin H, polyphyllin VI and polyphyllin VII with KEAP1.

| **Compound** | **Total score** |
| --- | --- |
| Polyphyllin H | 7.3251 |
| Polyphyllin VI | 4.7186 |
| Polyphyllin VII | 2.9061 |

 **Supplemental Figure 1. Effects of RPE and PPH on LPS-induced RAW 264.7 cells.** The effects of gradient concentrations of PPH (A) and PRE (B) on the viability of RAW 264.7 cells. ***p* < 0.01, ****p* < 0.001 (Student’s *t*-test, treatment vs. control group).

**Supplemental Figure 2. Effects of LPS on the viability of RAW 264.7 cells and HaCaT cells.** The effects of gradient concentrations of LPS on the viability of RAW264.7 cell (A) and HaCaT cells (B). ****p* < 0.001 (Student’s *t*-test, treatment vs. control group).

**Supplemental Figure 3. HPLC chromatogram of RPE.** Retention times of commercial saponin standards are shown in the table.

**Supplemental Figure 4.** The effect of ML285 on the activation status of Nrf2 in PPH treated-RAW 264.7 cells. Immunofluorescence staining of NRF2 in fixed RAW 264.7 cells treated with 0.125 µg/ml PPH and 0.125 µg/ml PPH + 5 µmol/L ML385 for 6 h, respectively. The nuclear regions were stained with DAPI. Bar = 10 µm (A). Relative abundance of NRF2 in nuclear regions quantified by using ImageJ software (B). **P* < 0.05, (Student’s *t*-test). Data were collected from three independent experiments. Error bars show standard deviation.


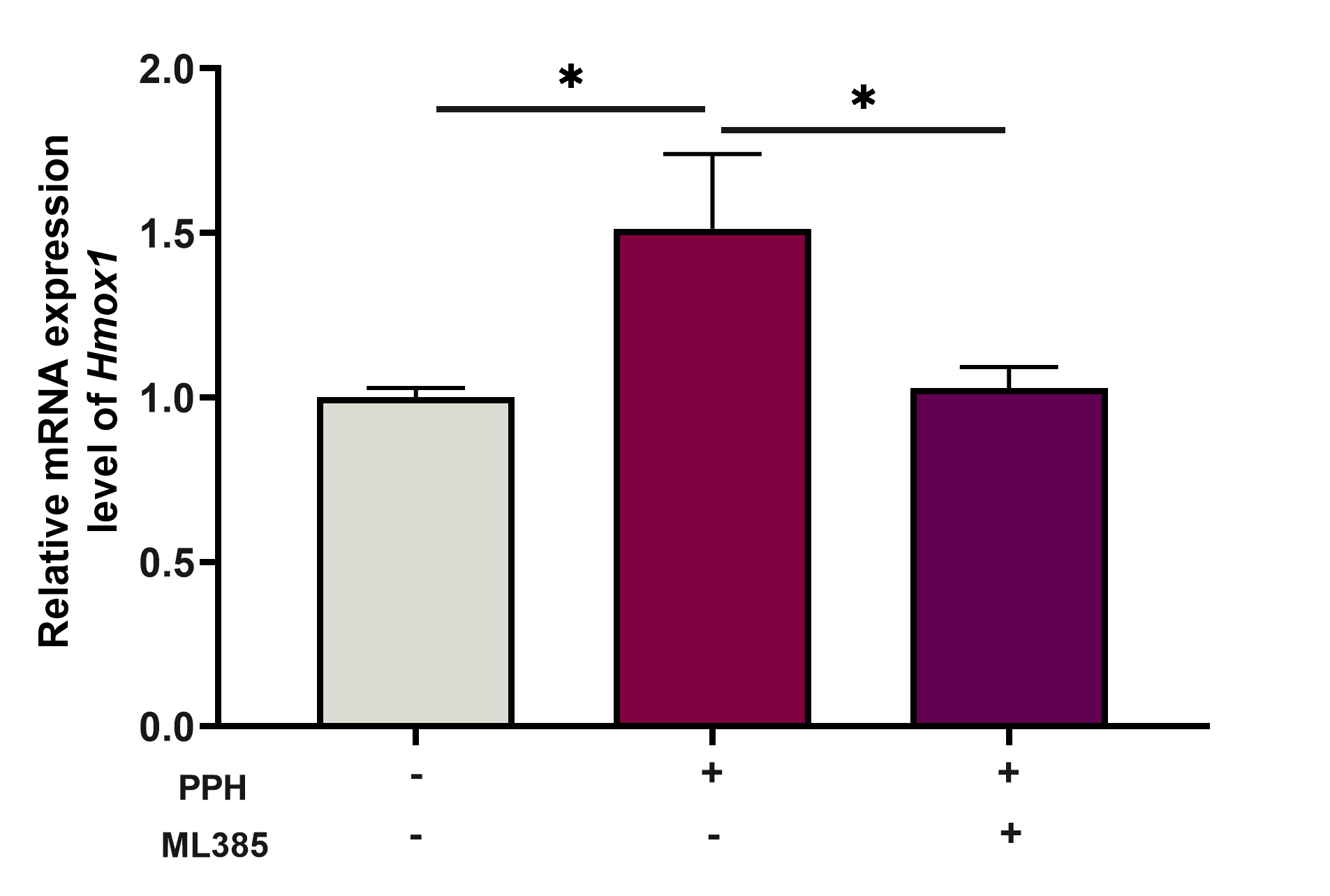


**Supplemental Figure 5. The effect of ML385 on the mRNA expression of *Hmox1* in PPH treated-RAW 264.7 cells.** *Gapdh* was used as internal control. RAW 264.7 cells were treated with 0.125 µg/ml PPH or the combination of 0.125 µg/ml PPH and 5 µmol/L ML385. **P* < 0.05, (Student’s *t*-test). Data were collected from three independent experiments. Error bars show standard deviation.

**Supplemental Figure 6. The effect of ML385 on intracellular ROS production in PPH treated-RAW 264.7 cells.** Fluorescence images of intracellular ROS generations in different groups (A). Bar = 100 µm. Fluorescence intensities of intracellular ROS assessed by ImageJ (B). Data were collected from three independent experiments. ***P* < 0.01 (Student’s *t*-test). Error bars show the standard deviation.
